# Preparation, Structure and Rheological Properties of Konjac Glucomannan–CaCl_2_ Electrogel

**DOI:** 10.3390/gels12060466

**Published:** 2026-05-28

**Authors:** Lixia Wang, Guorong Lin, Lijun Fu

**Affiliations:** 1 College of Environmental and Biological Engineering, Putian University, Putian 351100, China; ptlgrcub@163.com (G.L.); lijun_fu@sina.com (L.F.); 2 Key Laboratory of Ecological Environment and Information Atlas, Fujian Provincial University, Putian University, Putian 351100, China; 3 Fujian Key Laboratory of Ecological Impacts and Treatment Technologies for Emerging Contaminants, Putian University, Putian 351100, China

**Keywords:** konjac glucomannan (KGM), calcium chloride (CaCl_2_), alternating current (AC), electrogel, structure, rheological properties

## Abstract

The gelation property is one of the core functional characteristics of konjac glucomannan (KGM). KGM mainly forms gels through ionic crosslinking, deacetylation and compounding with other colloids. Exploring novel gelation technologies for the precise regulation of KGM gel properties is the research focus in this field. In this work, an alternating current (AC) electric field was applied to trigger KGM gelation in the presence of calcium chloride (CaCl_2_). The structure and viscoelastic properties (including storage modulus G′, loss modulus G″ and loss factor tanδ) of the gels were analyzed by Fourier transform infrared spectroscopy (FTIR), Raman spectroscopy (RS), scanning electron microscopy (SEM), X-ray diffraction (XRD), simultaneous differential scanning calorimetry/thermo-gravimetric analyzer (DSC/TGA) and rheometer. FTIR and RS revealed that KGM underwent partial degradation and deacetylation under the AC electric field. Calcium ions and chloride ions dissociated from CaCl_2_ are adsorbed onto the hydroxyl groups of KGM molecules. KGM molecules constituting the gels still retain partial original acetyl groups. SEM images showed that the gels had a porous structure with a coarse surface. XRD patterns showed the gels contained complex CaCl_2_ hydrates. Simultaneous DSC/TGA analysis indicated that the gel with excellent thermal stability exhibited distinct melting endothermic peaks corresponding to CaCl_2_ hydrates. Rheological data showed that, apart from KGM concentration, G′ and G″ of the gels gradually increased with the elevation of CaCl_2_ concentration, applied voltage and electric treatment duration. However, when CaCl_2_ concentrations, voltage, and electric treatment time exceeded their respective critical values, both started to decrease. Taking G′ as the evaluation index, the optimal preparation conditions for KGM-CaCl_2_ electrogel were determined as follows: KGM 0.5%, CaCl_2_ 1.2%, electric treatment duration 45 min, and voltage 24 V.

## 1. Introduction

Gels have been widely applied in new resource food products, food additives, food matrix, drug and nutrient carriers, tissue engineering scaffolds, packaging films, adhesives, etc. [1]. One of the major factors determining the gel performance is the preparation method and conditions, and gel properties further dictate its practical applications. There are many methods for preparing gels, such as heating, cooling, adjusting pH, mixing with other colloidal substances, ionic cross-linking, irradiation cross-linking, magnetic induction, etc. [2,3,4,5,6].

An electric field can also be applied to induce the formation of a gel and to control the gel structure for various applications. One typical application involves the use of low- voltage direct current (LVDC) for fabricating silk protein electrogels, which offers a promising strategy to encapsulate negatively charged proteins and gene drugs [7]. Under weak direct current (DC) electric fields, silk protein began to gel on the positive electrode. Interchain hydrogen-bonded metastable helical structures dominate the formation of silk electrogels, rather than *β*-sheet-mediated self-assembly [8,9]. Water electrolysis produces H^+^ and OH^–^ at the anode and cathode, respectively, causing localized pH variation. When the pH near the anode drops below the isoelectric point (pI), silk fibroin undergoes conformational transformation and further forms metastable electrogels [7]. In our previous studies, LVDC was used to induce the formation of KGM-sodium tungstate composite gels. The gelation mechanism indicated that isopolytungstate ions converted from WO_4_^2–^ could cross-link with the C-6 hydroxyl groups of KGM chains under DC electric field, thereby constructing a three-dimensional network structure [10]. However, when DC is applied to electrocoagulation or electrogelation, water electrolysis occurs, forming dense oxide layers on the cathode and inducing oxidative corrosion on the anode. The disadvantages of DC can be overcome by adopting low-amplitude alternating current [8,11]. Servoli et al. adopted alternating current (AC) electric fields (220 V, 50 Hz) to fabricate anisotropic silk fibroin films. The gel film formation mechanism was proven to be associated with molecular dipole alignment and the construction of oriented supramolecular assemblies [12].

KGM is a neutral polysaccharide isolated from *Amorphophallus konjac* tubers. It is composed of glucose and mannose units linked via *β*-1,4 glycosidic bonds, with a reported molar ratio of approximately 1:1.6. Side branches are attached to the main chain via *β*-(1 → 6) glucosyl linkages, and acetyl groups are periodically distributed along the backbone, which endows KGM with good water solubility [13]. It has been widely used in food, biomedical, environmental, bio-technical, and fine chemical industries, among others [14]. Owing to its unique structural characteristics, such as readily dissociable acetyl groups, electric field-sensitive glycosidic bonds and abundant polyhydroxyl structures [15,16], KGM is prone to structural and conformational changes under the combined effect of an AC electric field and metal ions, which will further alter its rheological behavior and gelation performance. So, to expand the application of KGM, we focused on the possibility of creating a new kind of gel of KGM under an AC electric field.

## 2. Results and Discussion

### 2.1. Formation of KGM-CaCl_2_ Electrogel Induced by AC Electric Field

When low-frequency and low-voltage AC (50 Hz) was applied to inert electrodes inserted into the KGM sol, no gel formed even after 45 min, and no heat release was observed. There were also no bubbles produced. But white aggregates were observed at the interface of gas and liquid when AC was applied to KGM sols containing a certain amount of CaCl_2_. The temperature of the sols increased, and bubbles were generated during the treatment. No gel formation was observed when the CaCl_2_ concentration ranged outside 0.6~1.4%, or when the voltage exceeded the range of 23~27 V. Meanwhile, we noticed that the degree of electrolytic corrosion in AC electrolysis was obviously much lower than that in DC electrolysis according to the abrasion of the inert electrodes [10,17]. The experimental phenomena are similar to those observed in the KGM-KCl electrogelation process [18]. Traditional KGM hydrogels are mainly formed by ionic crosslinking, alkali–thermal induction, and composite crosslinking. In this study, a low-frequency alternating electric field was used to induce gelation of KGM in the presence of Ca^2+^. Due to its formation at the air-liquid interface, the gel exhibits hydrophobic properties different from those of traditional bulk gels, showing great potential in oil adsorption and hydrophobic packaging membrane applications. This method has the advantages of simple operation and mild reaction conditions, which provide a new strategy and theoretical reference for the functional modification of KGM and the development and application of polysaccharide-based electrically responsive hydrogels.

### 2.2. FTIR Spectroscopic Analysis

**Figure 1:**
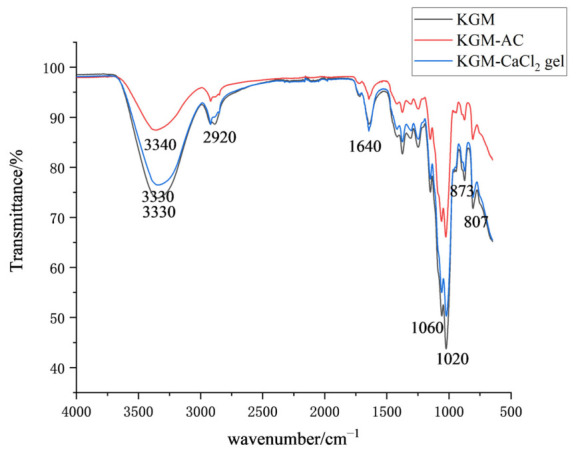
FTIR spectra of pure KGM powder, lyophilized KGM-AC (0.5% KGM, treated with 24 V AC for 20 min), and KGM-CaCl_2_ gel (0.5% KGM,1.2% CaCl_2_, treated with 24 V AC for 45 min).

Figure 1 shows the FTIR spectra of pure KGM powder, lyophilized AC-treated KGM sol (0.5% concentration, 24 V, 20 min, denoted as KGM-AC), and KGM-CaCl_2_ gel prepared with 0.5% KGM and 1.2% CaCl_2_ at 24 V for 45 min. Compared with KGM, KGM-AC and KGM-CaCl_2_ gels exhibited FTIR spectra similar to pristine KGM, with some differences accompanied simultaneously. KGM-AC displayed a weaker and broader peak at 3340 cm^−1^, demonstrating the perturbation of hydroxyl vibration modes. Meanwhile, the absorption peak of pristine KGM at 3330 cm^−1^ shifted toward higher wavenumbers in the KGM-AC sample. Appreciable shifts in the FTIR absorption bands meant distinct changes in chemical interactions (hydrogen bonding) between molecular chains [19,20]. It was also observed that the intensities of characteristic absorption peaks at 1720 cm^−1^, 1640 cm^−1^ and 1020 cm^−1^ declined, suggesting the cleavage of carbonyl groups and glycosidic bonds within KGM molecular chains [21,22]. Collectively, these results indicated that KGM underwent partial degradation and deacetylation under an AC electric field.

However, the peak intensities corresponding to the stretching vibrations at 3330 cm^−1^ (-OH), 1640 cm^−1^ (CH_3_COO-), and 1020 cm^−1^ (C-O-C) were restored in the FTIR spectrum of KGM-CaCl_2_ gel. The characteristic stretching peak of carbonyl groups at 1720 cm^−1^ indicated that partial acetyl groups were still retained in both KGM-AC and KGM-CaCl_2_ gel. These results are similar to those of KGM-KCl electrogels, and the underlying causes of these variations have also been explained in our previously published study [18].

### 2.3. Raman Spectroscopic Analysis

**Figure 2:**
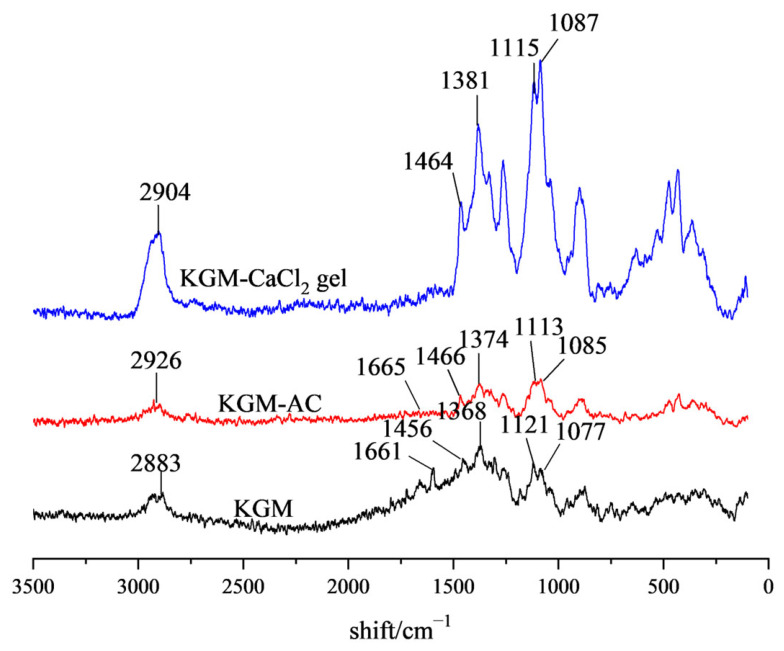
Raman spectra of native KGM, freeze-dried KGM-AC (0.5% KGM, 24 V, 20 min) and gel (0.5% KGM, 1.2% CaCl_2_, 24 V, 45 min).

Figure 2 presents the Raman spectra of pristine KGM, freeze-dried KGM sol (0.5% KGM, treated with 24 V AC for 20 min) and KGM-CaCl_2_ gel (0.5% KGM, 1.2% CaCl_2_, treated with 24 V for 45 min), which were recorded in the range of 0~3500 cm^−1^. Compared with native KGM pure powder, nearly all the typical characteristic peaks declined in the KGM-AC spectrum. The reductions in the vibration bands at 2926 cm^−1^ and 1665 cm^−1^ indicated that the C-H and C=O vibrational behaviors of KGM were obviously affected after AC treatment [23,24]. Meanwhile, the decreased peak intensities at 1113 cm^−1^ and 1085 cm^−1^ revealed the destruction of the C-O-C skeletal vibration and the breakage of glycosidic bonds [25]. These results suggested that KGM molecules degraded to a certain degree and were partially deacetylated after being treated by the AC electric field. However, these vibrational bands were significantly recovered in the Raman spectrum of KGM-CaCl_2_ gel as a result of electrostatic interactions. Moreover, the C-H bending and stretching vibration peaks of pristine KGM at 1456 cm^−1^ and 2883 cm^−1^ shifted to higher wave-numbers at 1464 cm^−1^ and 2904 cm^−1^ in KGM-CaCl_2_ gel, indicating conformation changes in hydrocarbon chains (symmetric and asymmetric vibrations of -CH) [26,27]. The shift in the KGM-CaCl_2_ gel was because the -CH_3_ or -CH_2_ of the KGM-CaCl_2_ gel was exposed to the polar environment of the CaCl_2_ solution [28]. The results are consistent with its FTIR spectral analysis and similar to the Raman spectra of KGM-KCl electrogels [18]. Combining with the observation that the gel formed at the gas–liquid interface, we propose that the main forces for the gel formation are electrostatic and hydrophobic interactions rather than hydrogen bonds.

### 2.4. SEM Observations

**Figure 3:**
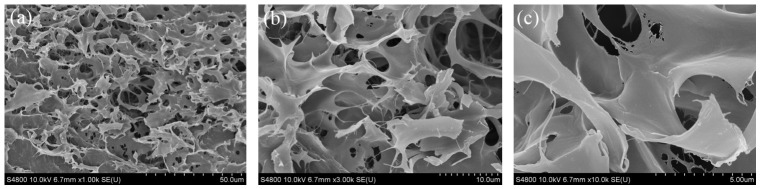
SEM images of lyophilized gels prepared with 0.5% KGM and 1.2% CaCl_2_ under 24 V for 45 min at different magnifications ((**a**) 1000×; (**b**) 3000×; (**c**) 10,000×).

Figure 3a–c displays SEM images of KGM-CaCl_2_ gels at different magnifications. It can be observed that the gels possessed a porous structure with inhomogeneous pore size distribution and coarse pore walls. The gels as a whole possessed a rough and irregular surface. It could be explained by the fact that the gel skeleton (KGM molecular chains) fractured during AC electric treatment. The fragmented short KGM chains failed to encapsulate water molecules and form a complete scaffold structure.

### 2.5. X-Ray Diffraction of KGM-CaCl_2_ Gels

**Figure 4:**
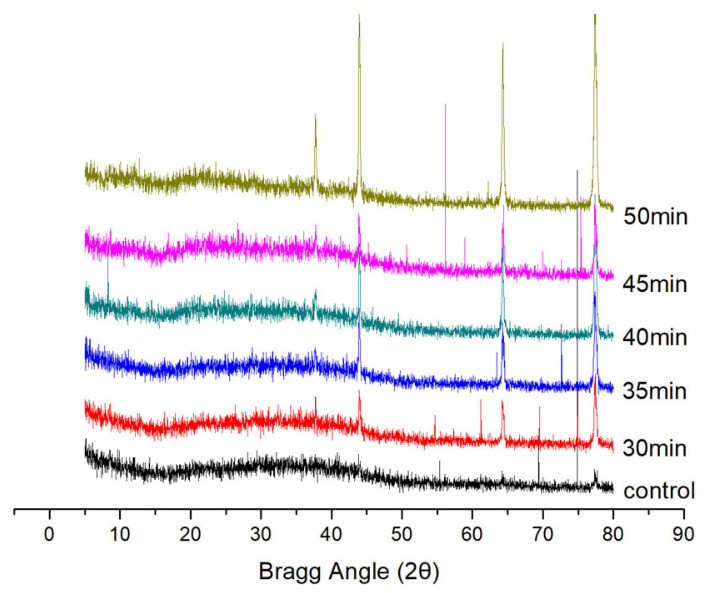
XRD patterns of lyophilized KGM-CaCl_2_ sol (control, 0.5% KGM, 0.6% CaCl_2_) and gels prepared at the same concentrations under 24 V for different treatment durations.

To clarify the influences of CaCl_2_ on the ordered structure and electrogel network of KGM, XRD patterns were collected from lyophilized KGM-CaCl_2_ sol (0.5% KGM, 0.6% CaCl_2_, control group) and KGM-CaCl_2_ gels fabricated via 24 V AC treatment for different durations. The curves are shown in Figure 4. As shown in the figure, only two weak diffraction peaks were observed at 2θ ≈ 77.4° and 64.2° in the XRD pattern of the control group. This demonstrates that CaCl_2_ exists predominantly in an amorphous state in the lyophilized KGM-CaCl_2_ composite system, with only a small fraction of CaCl_2_ present in crystalline form [29]. This could be explained by the fact that the CaCl_2_ dissociated in the KGM sol, and the released Ca^2+^ and Cl^–^ were adsorbed on the -OH groups of KGM. In addition, a small amount of undissociated CaCl_2_ molecules adsorbed on KGM molecular chains tends to absorb moisture easily after freeze-drying, thereby forming weak crystal diffraction peaks [30]. XRD characteristic peaks at 77.4°, 64.2°, 43.9° and 37.7° (2θ) of KGM-CaCl_2_ gels with different electric treatment durations were ascribed to complex calcium chloride hydrates, which originated from CaCl_2_ that was not adsorbed onto KGM molecules [29,31]. As reported by Eberbach et al., the specific pathways and products of the hydration/dehydration reactions of CaCl_2_ depend on reaction conditions (such as temperature and water vapor pressure), rather than following a single fixed reaction route. However, the confinement within the pore channels of porous materials alters these pathways: smaller pore diameters make it more difficult to form larger hydrate crystals [30]. In addition, it can be seen from the XRD pattern that the diffraction intensity of CaCl_2_ hydrates in the gels obtained with an electric treatment duration of 30~45 min is relatively low. This is because the gel structure formed at 30~45 min is denser, and CaCl_2_ hydrate is well encapsulated inside the gel network. In contrast, the gel obtained at 50 min shows a higher diffraction intensity of CaCl_2_ hydrate, which is attributed to the weaker gel structure at 50 min, resulting in insufficient encapsulation of CaCl_2_ hydrate. Moreover, the broad diffuse diffraction peak of KGM around 2θ ≈ 20° disappeared in both the lyophilized KGM-CaCl_2_ sol and KGM-CaCl_2_ gel systems, indicating a reduction in the ordered structure of KGM. The result is similar to the reported effect of KCl on the crystallinity of KGM [32]. This was attributed to the weakening of hydrogen bonds, and the decrease in hydrogen bonds within KGM-CaCl_2_ sols was caused by the occupation of partial hydroxyl groups on KGM molecules by Ca^2+^ and Cl^–^. Similar findings were reported by Chen et al., who demonstrated that the diffraction peak intensity of KGM decreased and its structural disorder increased following copper ion adsorption [33]. For KGM-CaCl_2_ gels, besides the binding of ions with the hydroxyl groups, the reduction of hydrogen bonds was also attributed to the AC-induced structural and conformational changes, as well as the penetration and entanglement of the fractured KGM molecular chains. In summary, although the AC electric field drives the entanglement of KGM molecules to form a three-dimensional network structure in the presence of Ca^2+^, it does not increase the crystallinity of KGM in the gel. The above results were similar to those of KGM-KCl electrogels [32].

### 2.6. DSC/TGA Analysis

**Figure 5:**
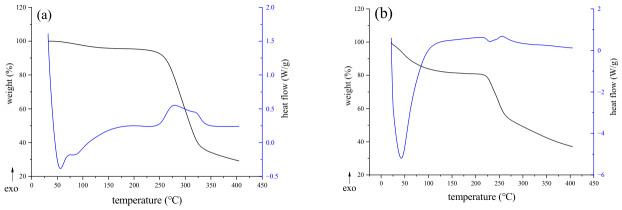
Simultaneous DSC-TGA curves of native KGM (**a**) and gel prepared with 0.5% KGM and 1.2% CaCl_2_ under 24 V for 45 min (**b**). (Note: Exo up indicates that exothermic peaks point upward.)

Simultaneous DSC-TGA measurements were performed to characterize the thermal behaviors of raw KGM powder and KGM-CaCl_2_ gel. The corresponding DSC/TGA curves are presented in Figure 5a,b. As shown in the TGA curves, both pristine KGM powder and KGM-CaCl_2_ gel exhibited three distinct weight loss stages. The first-stage weight loss below 225 °C of KGM-CaCl_2_ gel was higher than that of pristine KGM, which was attributed to the evaporation of bound water and the easier thermal degradation of fragmented KGM molecular chains in the gel structure. Meanwhile, a second sharp weight loss was observed between 225 °C and 250 °C, which originated from the thermal decomposition of the gel network. However, the second weight loss stage was shorter in duration than that of KGM. Moreover, the third-stage weight loss of the gel was lower than that of native KGM, which was attributed to the entangled KGM structures in the gel. When the temperature reached 225 °C, the weight loss of the gel was approximately 20%, indicating its favorable thermal stability.

As observed from the DSC curves, KGM presented an endothermic peak in the range of 45.7~110.9 °C and an exothermic peak at approximately 250~350 °C [34]. In contrast, the KGM-CaCl_2_ gel exhibited two distinct endothermic peaks, which were similar to those of the KGM-KCl electrogel, confirming the existence of crystalline substances in the KGM-CaCl_2_ gel [32,35]. The first intense endothermic peak near 50 °C was due to the superimposition of the melting endothermic peaks of calcium chloride hydrate and crystal water adsorbed in the gel pores [36]. The second weak endothermic peak at 200~250 °C was attributed to the melting of the gel structure, indicating that the gel possessed good thermal stability [37], which was in accordance with the analysis of TGA.

### 2.7. The Rheological Properties of KGM-CaCl_2_ Electrogels

#### 2.7.1. Effect of CaCl_2_ Concentration on G′, G″ and Tanδ of KGM-CaCl_2_ Electrogels

To explore the formation conditions of KGM-CaCl_2_ electrogels, the effects of four key factors, namely CaCl_2_ concentration, electric treatment duration, applied voltage and KGM concentration, on G′, G″ and tanδ of the gels were systematically investigated in sequence.

**Figure 6:**
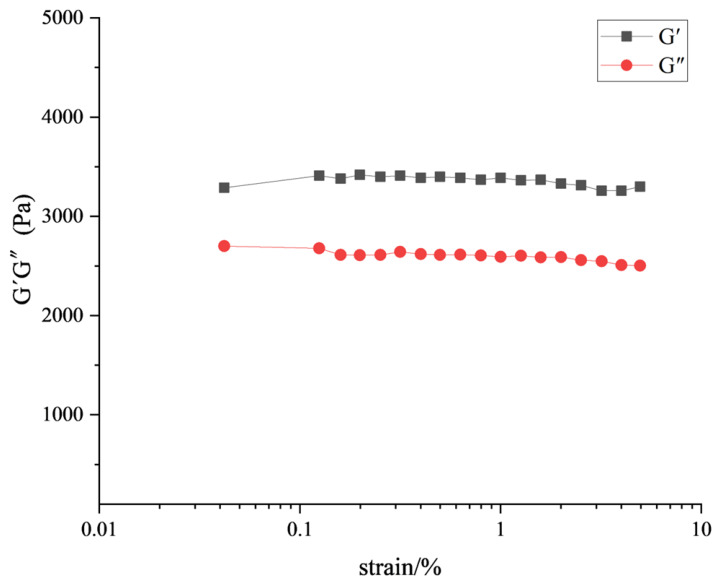
LVR of KGM-CaCl_2_ gel (0.5% KGM, 1.2% CaCl_2_, 24 V, 35 min).

First, strain amplitude sweep tests were carried out to determine the linear viscoelastic region (LVR) of KGM-CaCl_2_ gel. The relatively weaker gel prepared with 0.5% KGM, 1.2% CaCl_2_, 24 V voltage, and a 35 min electric treatment time was tested to ensure all samples were measured within the LVR. The results are presented in Figure 6. It can be seen that G′ and G″ of the gel remained stable below 1% strain, suggesting the gel network kept intact under small deformation. Accordingly, all frequency sweep tests were conducted at a fixed strain of 0.5%.

**Figure 7:**
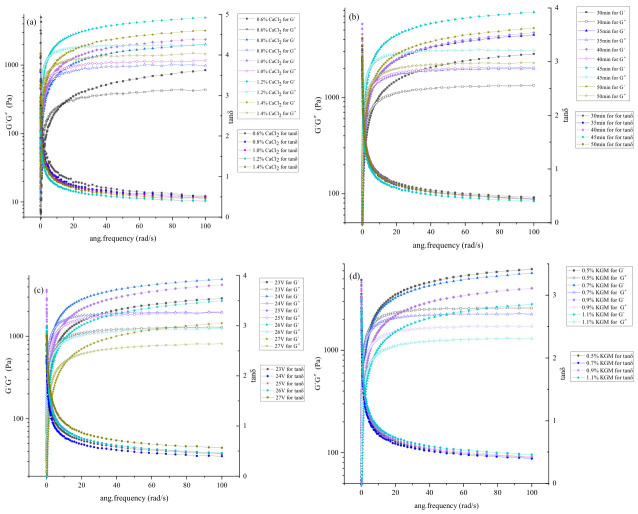
Frequency dependence of G′ (solid symbols), G″ (open symbols) and tanδ for KGM-CaCl_2_ gels ((**a**) different CaCl_2_ concentration, 0.5% KGM, 25 V, 40 min; (**b**) different electric treatment times, 0.5% KGM, 1.2% CaCl_2_, 25 V; (**c**) different applied voltage, 0.5% KGM, 1.2% CaCl_2_, 45 min; (**d**) different KGM concentration, 1.2% CaCl_2_, 45 min, 24 V).

Due to the important role of CaCl_2_ in gel formation, the effects of CaCl_2_ concentration on G′, G″ and tanδ of the gels were investigated. As shown in Figure 7a, in the low-frequency region, G′ was lower than G″, while G′ exceeded G″ in the high-frequency region, indicating the formation of a weak, hydrophobic association gel. This frequency-dependent response is characteristic of a dynamic network, where the reversible junctions can break and recombine under shear, which typically imparts shear-thinning and potential self-healing properties to the material [38]. Both G′ and G″ increased slightly at high frequencies, indicating the formation of a well-developed three-dimensional network within the gels. This mild increase in moduli further demonstrates the excellent deformation resistance of the gel systems [10,39]. By comparing the modulus variations at different CaCl_2_ concentrations, we observed that both G′ and G″ increased gradually with the elevation of CaCl_2_ concentration from 0.6% to 1.2%. Currently, the crossover point where tan δ = 1 has shifted to lower frequencies, indicating that higher ionic concentrations lead to the formation of a more robust gel network. This is because with the rise in CaCl_2_ content, more Ca^2+^ and Cl^–^ bind to the hydroxyl sites of KGM molecules [40]. The enhanced surface charge due to ion adsorption promotes the interpenetration of the charged KGM chains, thereby forming a stable network structure under the AC electric field. Furthermore, KGM sols with 0.6~1.2% CaCl_2_ exhibited boiling in the later period of AC treatment, and higher salt concentrations advanced the boiling time. Moderate boiling not only promoted gel formation at the gas–liquid interface but also expelled partial gases from the gel network. However, both G′ and G″ declined when the CaCl_2_ concentration exceeded 1.2%. Excessive addition of CaCl_2_ released more heat during AC electric treatment. Thus, the cumulative thermal effects not only accelerated the degradation of KGM molecular chains but also destroyed the structural stability of the preformed gel network. Once the CaCl_2_ concentration exceeded 1.4%, gels could no longer form. Excess CaCl_2_ caused violent boiling of the KGM sol during the treatment, which inhibited normal gel formation. The gelation process demonstrates that CaCl_2_ concentration is a key factor in regulating the formation and network structure of KGM-CaCl_2_ electrogels. Therefore, a CaCl_2_ concentration of 1.2% was selected for the subsequent experiments.

#### 2.7.2. Effect of Electric Treatment Duration on G′, G″ and Tanδ of KGM-CaCl_2_ Electrogels

Secondly, to clarify the effects of AC electric field treatment on the formation and network structure of KGM-CaCl_2_ electrogels, the influences of electric treatment duration on G′, G″ and tanδ of the gels were investigated. The relevant results are presented in Figure 7b. Gelation of KGM-CaCl_2_ commenced at an AC treatment time of 25 min, whereas no gels formed with a treatment time shorter than 25 min. A similar phenomenon was observed for KGM-KCl electrogels [18,32]. As illustrated in Figure 7b, both G′ and G″ increased gradually when the electric treatment duration was extended from 30 min to 45 min, with G′ dominating over G″. This is because under fixed voltage, CaCl_2_ and KGM concentrations, longer treatment time allowed sufficient interpenetration and entanglement of KGM molecular chains, thereby constructing a more compact gel network structure. When the electric treatment duration was extended from 45 min to 50 min, both G′ and G″ decreased correspondingly, suggesting a decline in gel strength. Further extending the treatment duration beyond 55 min caused the preformed gel to dissolve. This is because the entangled KGM molecular chains undergo disentanglement, depolymerization, and degradation under prolonged electric treatment and elevated temperature. This phenomenon is analogous to the behavior of KGM in KCl solution. Our previous studies have shown that the molecular weight and viscosity of KGM decrease significantly with the extension of electric treatment time in KCl solution [18,32]. These results further demonstrated the critical role of appropriate AC electric treatment duration in the formation of KGM-CaCl_2_ electrogels. Consequently, 45 min was chosen as the optimal electric treatment time for the following experiments.

#### 2.7.3. Effect of Voltage on G′, G″ and Tanδ of KGM-CaCl_2_ Electrogels

Thirdly, to investigate the effect of applied voltage on the gel formation and structure, the influences of voltage on G′, G″ and tanδ of the gels were investigated. The results are presented in Figure 7c. It can be seen from Figure 7c that voltage exhibited a remarkable impact on the moduli of the prepared gels. Even an increase of 1 V caused substantial changes in both G′ and G″. The voltage-dependent rheological behavior exhibited a similar trend to that induced by CaCl_2_ concentration and electric treatment duration. When the applied voltage increased from 23 V to 24 V, both G′ and G″ increased steadily, and G′ was always higher than G″. This was because the strengthened electric field accelerated the migration and entanglement of charged KGM molecular chains. Within a fixed treatment duration, the molecular chains achieved a higher degree of entanglement, thereby improving the mechanical strength of the resulting gel network. However, as the voltage rose from 24 V to 27 V, both G′ and G″ decreased correspondingly. This phenomenon can be explained from two aspects. First, a high electric field strength accelerated the migration of unadsorbed Ca^2+^ and Cl^–^, which generated substantial heat and weakened the structural stability of the gels. Second, high temperature combined with intense electric field action aggravated the fracture of the KGM molecular chains. When the voltage was lower than 23 V or higher than 27 V, no gelation occurred. This was because an excessively low current failed to drive the migration and entanglement of the charged KGM molecular chains. By contrast, an overly strong electric field and the accompanying excessive heat also inhibited the formation of the intact gel network. Furthermore, excessively high voltage induced violent boiling of the KGM-CaCl_2_ sol. The variations in moduli and gel formation behavior with applied voltage suggested that a suitable voltage is critical for the formation and structural stability of KGM-CaCl_2_ electrogels. Meanwhile, the results also reveal that an AC electric field is an indispensable prerequisite for the fabrication of the KGM-CaCl_2_ electrogel. Therefore, 24 V was determined as the optimal voltage for subsequent experiments.

#### 2.7.4. Effect of KGM Concentration on G′, G″ and Tanδ of KGM-CaCl_2_ Electrogels

Finally, the effects of KGM concentration on G′ and G″ of KGM-CaCl_2_ electrogels were investigated. The results are displayed in Figure 7d. It was observed that both G′ and G″ gradually declined as KGM concentration increased from 0.5% to 1.1%. This was because increased KGM concentration elevated the viscosity of KGM-CaCl_2_ sols, which hindered the movement, interpenetration, and crosslinking of KGM molecular chains. The effect of electric field strength on KGM sols containing a certain amount of CaCl_2_ was dominated by KGM degradation rather than driving the migration of KGM molecules. On the other hand, the decreased ratio of Ca^2+^ to KGM reduced the adsorption of Ca^2+^ ions onto the polymer chains. Consequently, the electrophoretic driving force under the AC electric field was diminished, leading to fewer collisions and weaker ion–dipole crosslinking between KGM chains. This resulted in a decrease in gel strength. When the KGM concentration reached 1.3%, the sol exhibited excessively high viscosity, which severely restricted the mobility of KGM molecules. Accordingly, gels could not be formed at this concentration. The dependence of G′ and G″ of KGM-CaCl_2_ electrogels on KGM concentration exhibited a trend similar to that of KGM-tungsten gels, but opposite to that of KGM-KCl electrogels [10,18]. This might be attributed to the much higher hydration enthalpy of CaCl_2_ compared with KCl [41], which hinders the adsorption of Ca^2+^ onto KGM molecular chains. However, considering that the gel yield increased with the rise in KGM concentration, 1.1% could also be chosen as an appropriate concentration for preparing KGM-CaCl_2_ electrogels. In summary, the optimized parameters for gel preparation are shown in Table 1.

### 2.8. Gelation Mechanism of KGM-CaCl_2_ Electrogel

**Figure 8:**
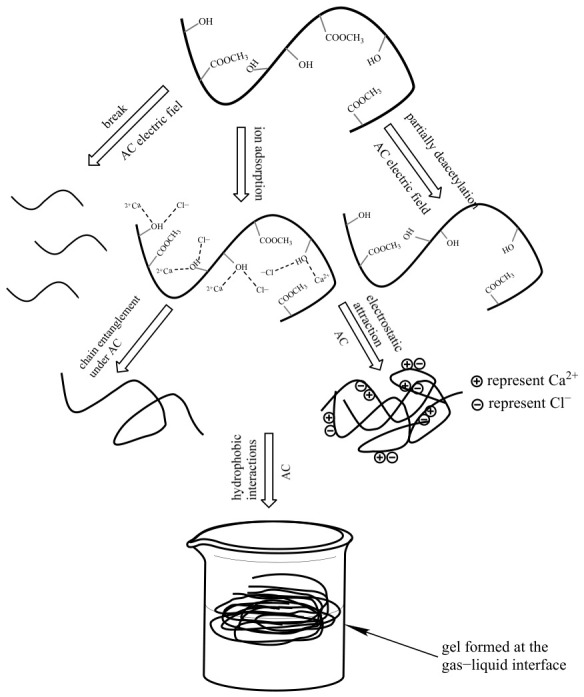
Schematic illustration of the formation mechanism of KGM-CaCl_2_ electrogel.

Based on the above experimental results and analysis, the gelation mechanism of KGM-CaCl_2_ electrogel was summarized as follows: first, under AC electric treatment, KGM molecular chains undergo a certain degree of scission and partial deacetylation. Meanwhile, Ca^2+^ and Cl^–^ dissociated from CaCl_2_ adsorb onto the -OH sites of KGM molecules, endowing the molecular chains with charge properties. Second, driven by AC, the charged KGM molecular chains aggregate via electrostatic interactions and interpenetrate with each other, forming entanglement structures between the two electrodes. The breakage of KGM molecular chains increases the quantity of intertwined molecules per unit volume. Finally, hydrophobic interactions drive KGM molecular chains to form a gel at the gas–liquid interface. Its gelation mechanism is different from that of KGM–titanium, KGM–boron, and KGM–sodium tungstate gels formed by ionic crosslinking. Their gelation mechanisms lie in that titanium ions released from organotitanium crosslinkers, borate ions dissociated from borates, and tungstate ions dissociated from sodium tungstate can separately crosslink with the hydroxyl groups on KGM molecular chains, thus forming a three-dimensional gel network structure [10,42,43]. The mechanism diagram of KGM-CaCl_2_ electrogel is shown in Figure 8.

## 3. Conclusions

In this work, we successfully fabricated KGM-CaCl_2_ electrogels via alternating current. The structure and rheological properties of the gels were investigated, and the underlying gelation mechanism was proposed. The results showed that KGM molecular chains were partially degraded and deacetylated during AC electric treatment. The gels retained part of the inherent characteristic acetyl groups of KGM, exhibiting an inhomogeneous porous structure with a rough surface. In addition, the gels contained complex CaCl_2_ hydrates and showed favorable thermal stability. The storage and loss moduli increased with increasing CaCl_2_ concentration, applied voltage, and electric treatment duration, but decreased with the increasing of KGM concentration; nevertheless, both moduli declined once the former three variables exceeded their respective critical values. Single-factor experiments indicated that the optimal conditions for gel preparation were 0.5% KGM, 1.2% anhydrous CaCl_2_, and 45 min of electric treatment at 24 V. The gelation mechanism could be described as follows: KGM interacts with CaCl_2_ to construct an ion–hydrogen bridging structure of 
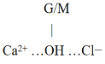
. Under an AC electric field, the charged KGM molecular chains interpenetrate and entangle mutually, ultimately assembling into a stable gel network. Distinct from conventional KGM gels, this gel exhibits notable hydrophobicity, suggesting potential applications in oil adsorption and hydrophobic film fabrication. In future studies, the effects of acetyl content, KGM molecular weight, and electrode distance on the formation, structure, and properties of the gel should be investigated. The successful fabrication of this gel further supports the formation mechanism of AC-induced KGM gels. Meanwhile, its structural characteristics and viscoelastic moduli are distinct from those of KGM-KCl electrogels, which may endow it with unique application potential compared with KGM-KCl electrogels.

## 4. Materials and Methods

### 4.1. Materials

Konjac pure powder containing 91.4% KGM was obtained from Sanai Konjac Biotechnology Co., Ltd., Zhaotong, China. Anhydrous CaCl_2_ (analytical grade) was purchased from Cuilin Huabo Instrument Co., Ltd., Zhangzhou, China.

### 4.2. Preparation of KGM-CaCl_2_ Electrogels Under AC Electric Field

Firstly, KGM hydrosol with concentrations of 0.5~1.1% was prepared, and anhydrous CaCl_2_ was added at a dosage of 0.6~1.4%. The mixture was fully shaken and homogenized. Subsequently, 50 g of the well-mixed KGM-CaCl_2_ sol was directly weighed in a 100 mL beaker. A perforated epoxy resin board was covered on the beaker, and the distance between the two holes was 4.0 cm. Two inert electrodes were inserted into the sol through the holes. An alternating current power supply (50 Hz) was connected, with an applied voltage of 23~27 V and a frequency of 50 Hz. After a period of energization, the KGM-CaCl_2_ electrogel was formed.

### 4.3. FTIR

FTIR spectra were recorded on a Nicolet iS10 FTIR spectrometer (Thermo Fisher Scientific Inc., Waltham, MA, USA) in the range of 400~4000 cm^−1^ in ATR mode. The instrumental resolution was 0.5 cm^−1^, and the number of scans was 32.

### 4.4. Raman

Raman spectra were acquired using a Labram Aramis confocal microprobe (Horiba Jobin-Yvon, Longjumeau, France) fitted with an Olympus microscope objective and a CCD cooled detector.

### 4.5. SEM

The surface morphology of KGM-CaCl_2_ was characterized using a Hitachi S-4800 cold field-emission scanning electron microscope (Hitachi, Ltd., Tokyo, Japan). Prior to observation, the samples were mounted on aluminum substrates with double-sided conductive carbon adhesive tapes, followed by gold sputtering for 90 s at a sputtering current of 10 mA. The SEM observations were conducted at an accelerating voltage of 15 kV, working distance of 6.9 mm, and emission current of 10.1 mA.

### 4.6. XRD

XRD patterns were acquired using an XRD-6100 diffractometer (Bruker, Ettlingen, Germany) equipped with Cu Kα radiation operated at 40 kV and 30 mA. Measurements were performed in the 2θ range 5~80° at a scanning rate of 2° min^−1^.

### 4.7. Simultaneous DSC/TGA

The thermal properties of the materials were characterized using a Q600 simultaneous TGA/DSC instrument (TA Instruments, New Castle, DE, USA). The samples were heated from 30 to 400 °C at a rate of 10 °C/min in an alumina crucible, with each sample weighing approximately 3~6 mg. A nitrogen purge flow of 100 mL/min was maintained throughout the measurements.

### 4.8. Rheological Measurements

Rheological measurements were performed using an AR-2000ex stress-controlled rheometer (TA Instrument, New Castle, DE, USA) equipped with a Peltier temperature controller. A parallel-plate geometry (20 mm diameter, 0.2 mm gap) was used. All samples were equilibrated at 25 °C for 2 min prior to frequency sweep measurements, and all tests were performed at a constant strain of 0.5% [10,18,44].

## Figures and Tables

**Table 1:** The optimized parameters for gel preparation.

CaCl_2_ Concentration (%)	Electric Treatment Duration (min)	Voltage (V)	KGM Concentration (%)	Storage Modulus (G′, Pa)
1.2	45	24	0.5	6~7 × 10^3^

## Data Availability

The data presented in this study are available upon request from the corresponding author.
